# Tetracycline Analogs Inhibit Osteoclast Differentiation by Suppressing MMP-9-Mediated Histone H3 Cleavage

**DOI:** 10.3390/ijms20164038

**Published:** 2019-08-19

**Authors:** Yeojin Kim, Jinman Kim, Hyerim Lee, Woo-Ri Shin, Sheunghun Lee, Jisu Lee, Jae-Il Park, Byung Hak Jhun, Yang-Hoon Kim, Sun-Ju Yi, Kyunghwan Kim

**Affiliations:** 1 School of Biological Sciences, College of Natural Sciences, Chungbuk National University, Cheongju, Chungbuk 361-763, Korea; 2 Asan Medical Center, Asan Institute for Life Sciences, Seoul 05505, Korea; 3 Korea Basic Science Institute, Gwangju Center at Chonnam National University, Gwangju 500-757, Korea; 4 Department of Cogno-Mechatronics Engineering, Pusan National University, Busan 46241, Korea

**Keywords:** tigecycline, minocycline, MMP-9, osteoclast, histone cleavage, osteoporosis

## Abstract

Osteoporosis is a common disorder of bone remodeling, caused by the imbalance between bone resorption by osteoclasts and bone formation by osteoblasts. Recently, we reported that matrix metalloproteinase-9 (MMP-9)-dependent histone H3 proteolysis is a key event for proficient osteoclast formation. Although it has been reported that several MMP-9 inhibitors, such as tetracycline and its derivatives, show an inhibitory effect on osteoclastogenesis, the molecular mechanisms for this are not fully understood. Here we show that tetracycline analogs, especially tigecycline and minocycline, inhibit osteoclast formation by blocking MMP-9-mediated histone H3 tail cleavage. Our molecular docking approach found that tigecycline and minocycline are the most potent inhibitors of MMP-9. We also observed that both inhibitors significantly inhibited H3 tail cleavage by MMP-9 in vitro. These compounds inhibited receptor activator of nuclear factor kappaB ligand (RANKL)-induced osteoclast formation by blocking the NFATc1 signaling pathway. Furthermore, MMP-9-mediated H3 tail cleavage during osteoclast differentiation was selectively blocked by these compounds. Treatment with both tigecycline and minocycline rescued the osteoporotic phenotype induced by prednisolone in a zebrafish osteoporosis model. Our findings demonstrate that the tetracycline analogs suppress osteoclastogenesis via MMP-9-mediated H3 tail cleavage, and suggest that MMP-9 inhibition could offer a new strategy for the treatment of glucocorticoid-induced osteoporosis.

## 1. Introduction

Bone is a rigid but dynamic organ in which remodeling occurs continuously throughout life. The remodeling process is tightly controlled by bone-forming osteoblasts and bone-resorbing osteoclasts. An imbalance between bone resorption and bone formation can cause a variety of skeletal disorders, such as osteoporosis, osteopetrosis, and Paget’s disease of bone [1]. Osteoclasts are giant, multinucleated cells that differentiate from hematopoietic progenitors of myeloid lineage; they function to resorb bone, removing old or damaged bone matrix. The receptor activator of the receptor activator of nuclear factor kappaB ligand (RANKL), a membrane-bound protein found in osteoblasts and osteocytes, is necessary for the differentiation of osteoclast precursors into osteoclasts [2]. The binding of RANKL to its receptor RANK on pre-osteoclast cell membranes leads to the activation of distinct signaling cascades, followed by the auto-amplification of NFATc1, a master transcription factor for osteoclast differentiation. NFATc1 up-regulates the expression of genes required for osteoclast maturation, such as *Trap*, *Cathepsin K*, and *Mmp-9* [3].

The matrix metalloproteinases (MrMPs) are a family of zinc-dependent endopeptidases that are known to play a key role in extracellular matrix remodeling. A total of 23 MMPs have been identified in humans. All MMPs share several conserved domains, including a pre-domain for protein secretion, a pro-domain for the regulation of enzyme activity, a zinc-containing catalytic domain, and a hemopexin domain for interaction with substrates. Notably, MMP-2, MMP-9, MMP-13, MMP-14, and MMP-16 have been implicated in bone development, remodeling, and repair, as well as in degrading the extracellular matrix [4]. In particular, MMP-9 expression is up-regulated during osteoclast differentiation, which subsequently stimulates bone resorption [5]. Our recent study unexpectedly showed that MMP-9 was observed in the nuclei of mouse bone marrow-derived macrophages (BMMs), where the nuclear MMP-9 cleaves the histone H3 N-terminal tail (H3NT) during osteoclast differentiation and regulates osteoclastogenic gene transcription through histone H3 proteolysis [6]. We also revealed that CREB binding protein/p300-mediated H3K18 acetylation and G9-mediated H3K27 monomethylation are necessary for MMP-9 to function as a protease, cleaving the H3 N-terminal tail of osteoclastogenic genes [7]. In this regard, MMP-9 plays a critical role in the active transcription of osteoclastogenic genes via H3 N-terminal tail cleavage under tight epigenetic regulation.

Glucocorticoids are widely used to suppress inflammation in a variety of chronic inflammatory diseases, including rheumatoid arthritis and inflammatory bowel diseases, and are also used as immunosuppressive agents, both for patients with malignancies and for organ transplant recipients; however, the glucocorticoids show diverse side effects [8]. In addition, they exert effects on bone and muscle via several mechanisms, including a decrease in the number and function of osteoblasts and an increase in the life span and differentiation of osteoclasts. Long-term glucocorticoid therapy has been linked to glucocorticoid-induced osteoporosis (GIO). GIO is a common cause of secondary osteoporosis, and increases the risk of bone fractures [8]. Bisphosphonates, anti-resorptive drugs, are considered to be first-line therapeutic options for GIO [9]. Since the effects of the long-term use of bisphosphonates on GIO remains unclear, and because bisphosphonates have a prolonged half-life and can cross the placenta, possibly leading to adverse effects on fetal bone development, the use of bisphosphonates for women of childbearing age should be carefully considered [8,10].

Tetracyclines are a family of broad spectrum antibiotics that share a common basic structure—a linear fused tetracyclic nucleus—to which a variety of functional groups may be attached [11]. First-generation tetracyclines (e.g., chlortetracycline, oxytetracyline, and tetracycline) were isolated from some *Streptomyces* species in the mid-1950s. To generate more potent tetracyclines with higher activity, second-generation semisynthetic tetracyclines (e.g., doxycycline and minocycline) were produced by chemically modifying the first-generation tetracyclines [11]. Recently, third-generation tetracyclines, such as tigecycline, have been developed to overcome bacterial resistance to the earlier compounds [12]. In addition to their antimicrobial activity, tetracyclines have also been reported to block bone resorption [13,14]. For example, doxycycline and minocycline have been shown to have inhibitory effects on osteoclastogenesis by blocking MMP-9 activity [15]. Tigecycline, a derivative of minocycline, is the first of the novel glycylcycline class; it has a broad spectrum of activity against both Gram-positive and Gram-negative bacteria, and can avoid many of the common antibiotic-resistance mechanisms that inactivate other tetracyclines [11,12]. Tigecycline is widely used to treat skin-structure infections and complicated intra-abdominal infections [16]. Like other tetracyclines, tigecycline, besides its antimicrobial activity, exhibits additional pharmacological properties, such as accelerating wound healing in staphylococcal-infected burns, as well as anti-tumor effects [17,18]. However, the effect of tigecycline on bone formation or bone resorption has yet to be investigated.

In the present study, our combined biochemical and computational studies revealed that tigecycline and minocycline were the most potent inhibitors for MMP-9. We showed that tigecycline and minocycline block the expression of osteoclast-specific genes by suppressing MMP-9-mediated H3 N-terminal cleavage during osteoclast differentiation, resulting in the inhibition of osteoclastogenesis. Furthermore, treatment with tigecycline and minocycline rescued the osteoporotic phenotype in a prednisolone-induced, osteoporosis zebrafish model.

## 2. Results

### 2.1. Tigecycline and Minocycline Suppress MMP-9-Mediated H3 Tail Cleavage

**Figure 1:**
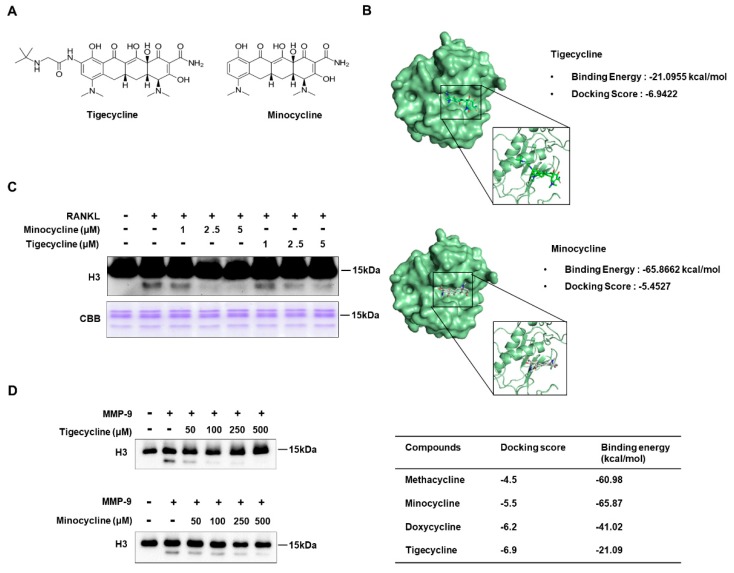
Tigecycline and minocycline inhibit histone H3 tail cleavage by matrix metalloproteinase-9 (MMP-9). (**A**) Chemical structures of tigecycline and minocycline. (**B**) Molecular docking analysis of MMP-9 and tetracycline derivatives. (**C**) Bone marrow-derived macrophages (BMMs) were treated with macrophage colony-stimulating factor (M-CSF) and receptor activator of nuclear factor kappaB ligand (RANKL) in the absence or the presence of tigecycline or minocycline for three days. Chromatin fractions were prepared and subject to Western blot analysis using an anti-H3 antibody (upper panel) and Coomassie brilliant blue staining (lower panel). (**D**) Inhibitory effects of tigecycline and minocycline on MMP-9-mediated H3 tail cleavage. An in vitro H3NT cleavage assay was performed using recombinant histone H3 and MMP-9, pre-incubated with or without tigecycline or minocycline at the indicated concentrations. The extent of H3 tail proteolysis was evaluated by Western blotting with the H3 antibody.

We recently demonstrated that the matrix metalloproteinase MMP-9 is the key protease involved in histone H3 tail cleavage during osteoclast differentiation [6]. Since tetracycline analogs are known to act as MMP-9 inhibitors [19,20], studying their possible effects on MMP-9-mediated H3 tail proteolysis was a logical extension of our study. To this end, we first attempted to identify the optimal inhibitor(s) of MMP-9 from the four tetracyclines currently in use. Our molecular docking and binding free-energy analysis revealed that minocycline and tigecycline had the lowest binding free energy (−65.87 kcal/mol) and the highest docking score (−6.9422), respectively (Figure 1A,B, and Figure S1). Therefore, these two inhibitors were selected for our study, based on the molecular docking analysis. We next determined whether these tetracycline analogs could inhibit MMP-9-mediated H3 tail cleavage during RANKL-induced osteoclast differentiation. To do this, osteoclast precursor (OCP) cells were treated with RANKL in the presence or absence of minocycline and tigecycline, in doses of 1, 2.5, or 5 μM. After purifying the chromatin fractions, histone H3 tail cleavage was determined by Western blotting. In agreement with our previous data [6], a fast-migrating H3 band, representing H3 N-terminal tail cleavage, was clearly detected following treatment with RANKL. However, the treatment of RANKL-induced osteoclasts with two MMP-9 inhibitors led to the pronounced inhibition of H3NT proteolysis (Figure 1C). The ex vivo results were further confirmed by an in vitro H3NT cleavage assay using these two inhibitors. Although both inhibitors significantly inhibited MMP-9-mediated H3 proteolysis in a dose-dependent manner (Figure 1D), their half-maximal inhibitory concentration (IC50) was much higher in vitro than ex vivo. Peptide-chip analysis using fluorescence-conjugated H3 peptide substrates confirmed that minocycline blocked H3 tail proteolysis by MMP-9 (Figure S2).

### 2.2. Tigecycline and Minocycline Inhibit Osteoclastogenesis by Negatively Regulating Osteoclast-Specific Genes

**Figure 2:**
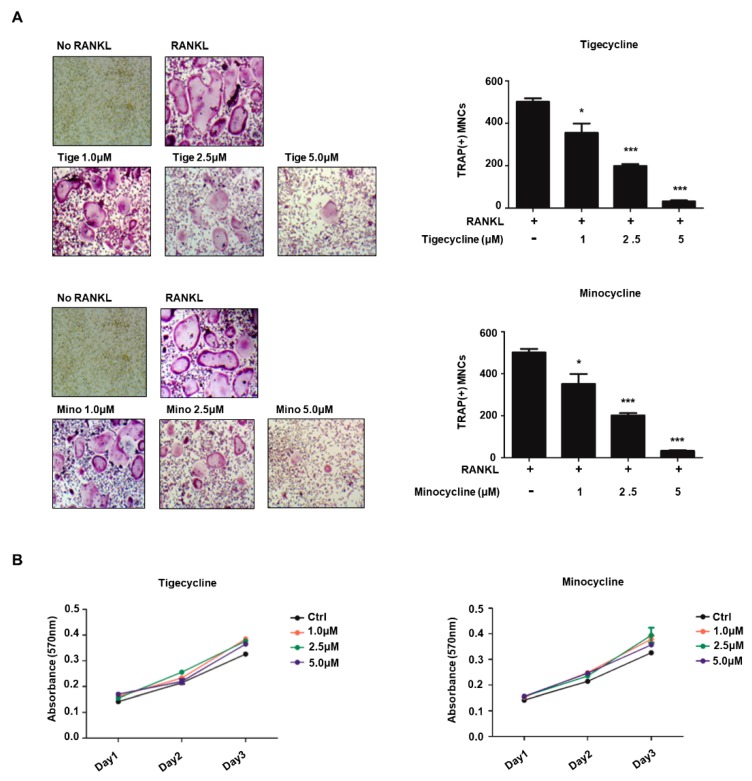
Tigecycline and minocycline suppress osteoclast formation from BMMs. (**A**) BMMs were treated with M-CSF and RANKL in the absence or the presence of increasing concentrations of tigecycline or minocycline for three days, and osteoclast formation was examined by tartrate-resistant acid phosphatase (TRAP) staining (left) and counting the number of TRAP-positive multinuclear osteoclasts (right). (**B**) BMMs were treated with M-CSF in the presence of tigecycline or minocycline, as in (**A**), and cell proliferation was measured by MTT assays. Error bars represent the mean result ± SD of three independent experiments; * *p* < 0.05, *** *p* < 0.0001 (ANOVA analysis).

We next investigated whether tigecycline and minocycline affect bone cell differentiation. We first examined the effects of tigecycline and minocycline on osteoclast formation in BMMs. Osteoclast precursors differentiated into tartrate-resistant acid phosphatase (TRAP)-positive, multinucleated osteoclasts following RANKL treatment. Treatment with the two tetracycline analogs significantly suppressed RANKL-induced osteoclast formation from the BMMs (Figure 2A). Given that osteoclast differentiation involves several critical stages, we also assessed the impact of tigecycline and minocycline on OCP cell proliferation. Exponentially growing BMMs, in the presence of macrophage colony-stimulating factor (M-CSF), were cultured with or without tigecycline and minocycline. MTT assays over a three-day time course revealed that tigecycline and minocycline did not affect the proliferation of osteoclast precursors (Figure 2B). These results suggest that the tetracycline analogs inhibit the differentiation but not the proliferation of pre-osteoclasts.

**Figure 3:**
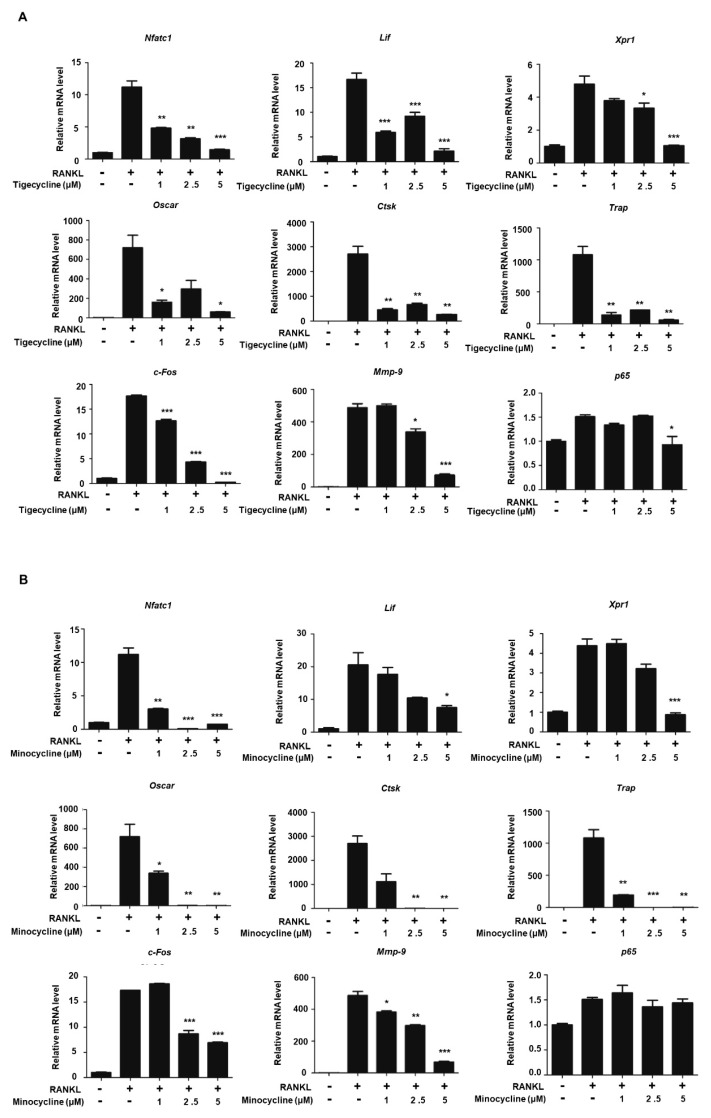
Effects of tigecycline and minocycline on the expression of osteoclast-related genes. (**A**,**B**) BMMs were treated with M-CSF and RANKL in the absence or presence of tigecycline or minocycline, as in Figure 2A. Total RNA was prepared from cells, and the expression levels of the nine osteoclast-related genes, as indicated, were assessed by qRT-PCR. Results represents the means ± SD of three independent experiments. * *p* < 0.05, ** *p* < 0.001, *** *p* < 0.0001 versus only RANKL treatment.

Our recent studies showed that MMP-9 directly transactivates a set of osteoclastogenic genes, including *Nfatc1*, *Lif*, and *Xpr1*, by facilitating H3NT cleavage [6]. Given that the two tetracycline analogs efficiently impaired MMP-9 proteolytic activity toward the H3NT, we also examined the role of these compounds with respect to the RANKL-induced expression of osteoclast-specific genes. BMMs were treated with tigecycline or minocycline in the presence of RANKL, and the expressions of several osteoclastogenic genes were evaluated by RT-PCR analysis. As shown in Figure 3A, RANKL treatment enhanced the expression of MMP-9 target genes, including *Nfatc1*, *Lif*, and *Xpr1*. As expected, tigecycline significantly repressed the RANKL-induced mRNA expression of the MMP-9 target genes in a dose-dependent manner (Figure 3A). We also observed that the expressions of Nfatc1 target genes (*Ctsk*, *Trap*, and *Oscar*) were significantly inhibited by tigecycline treatment. Similarly, we saw inhibitory regulation of osteoclast-specific genes following treatment with minocycline (Figure 3B). These results strongly suggest that the tetracycline analogs inhibit osteoclast differentiation by modulating MMP-9-mediated H3NT proteolysis.

### 2.3. Tigecycline and Minocycline Do Not Affect Osteoblast Differentiation

**Figure 4:**
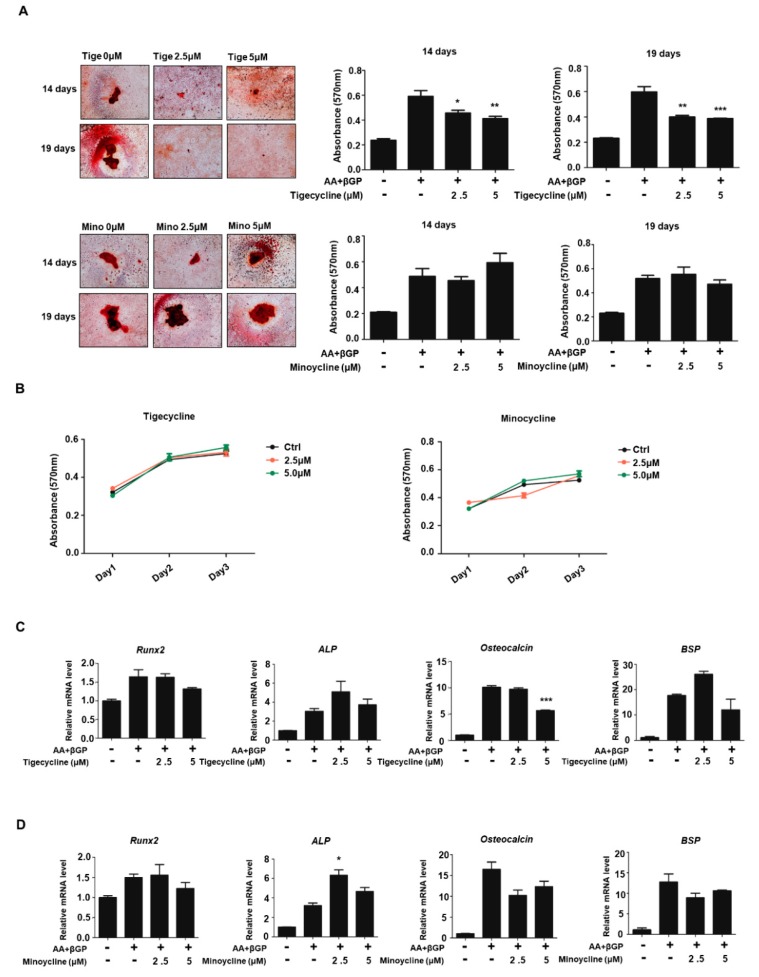
Effect of tigecycline and minocycline on osteoblast formation. (**A**) Primary osteoblasts from mouse calvaria were cultured in ascorbate (AA) and β-glycerophosphate (β-GP), with or without tigecycline or minocycline as indicated, for 14 or 19 days, and the Alizarin red S (ARS) staining for osteoblast differentiation was performed (left panel) and further quantified (middle and right panels). (**B**) Primary osteoblasts were treated with minocycline or tigecycline for three days, and cell proliferation was measured by MTT assays. (**C**,**D**) Primary osteoblasts were treated with or without tigecycline and minocycline in the differentiation medium, as in (**A**), for four days. Total RNA was prepared, and qRT-PCR was performed using primers specific for *Runx2*, *ALP*, *Osteocalcin*, and *BSP* genes. The mRNA levels were normalized to β-actin control. The results shown are mean values from three independent experiments. Error bars represent the mean result ± SD of three independent experiments; * *p* < 0.05, ** *p* < 0.001, *** *p* < 0.0001 (ANOVA analysis), versus only ascorbate and β-glycerophosphate treatment.

To investigate the effect of tigecycline and minocycline on osteoblast formation, we first established osteoblast differentiation using primary osteoblast cells isolated from mouse calvaria. As shown in Figure 4A, treatments with ascorbate and β-glycerophosphate for 14 and 19 days increased mineralization levels, as assessed by Alizarin red S (ARS) staining. The addition of tigecycline decreased mineralization levels to some extent, whereas minocycline treatment had little effect on calcium deposition. We further assessed the impact of tigecycline and minocycline on osteoblast cell proliferation. We observed that these compounds had no effect on the proliferation of preosteoblasts (Figure 4B). To further determine whether the tetracycline analogs affect osteoblast-related genes (*Runx2*, *ALP*, *Osteocalcin*, *BSP*, etc.) during osteoblastogenesis, gene expressions were analyzed by RT-PCR analysis. We observed that four-day induction in osteoblast differentiation medium apparently increased the osteoblast-related markers. Although tigecycline and minocycline somewhat differentially regulated osteoblast gene expressions, it seems that these compounds did not significantly affect osteogenic gene expression (Figure 4C,D).

### 2.4. Tigecycline and Minocycline Suppress Prednisolone-Induced Osteoporosis in Zebrafish Larvae

**Figure 5:**
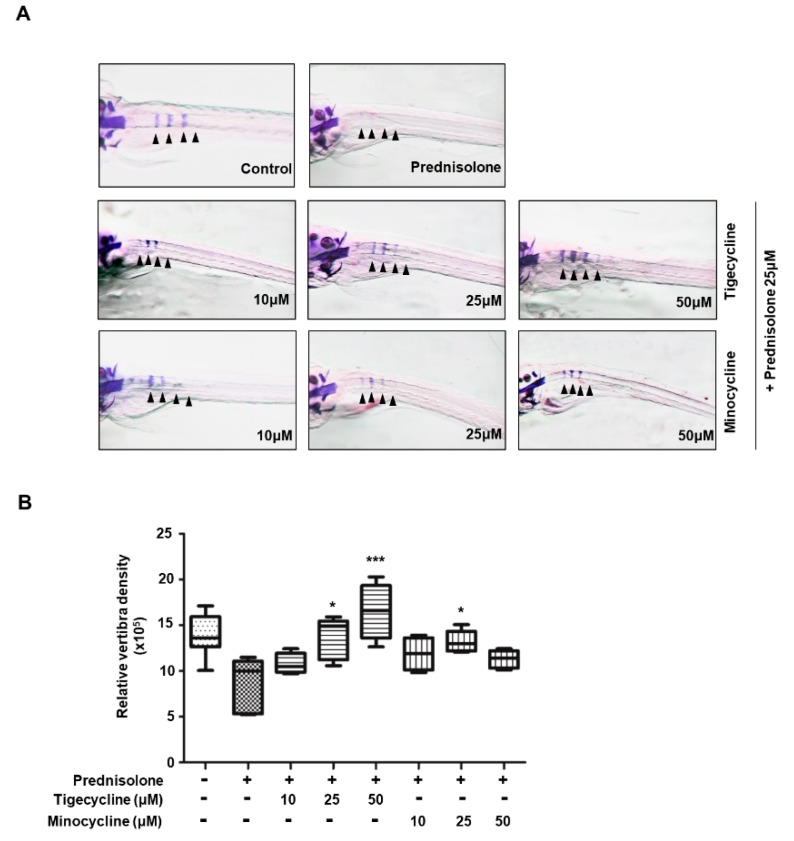
Anti-osteoporotic effect of tigecycline and minocycline in a model of prednisolone-induced osteoporosis in zebrafish. (**A**) The larvae at 10 dpf (days post-fertilization) were treated with 25 μM prednisolone in the presence or absence of tigecycline or minocycline, at the indicated concentrations. Whole-mount Alizarin red staining was performed to analyze the mineralized bone (arrowheads = bone Alizarin red staining). (**B**) Relative bone mineral density was calculated by measuring the areas of the first four stained vertebrae (V1–V4, indicated by arrowhead in (**A**)). * *p* < 0.05, *** *p* < 0.0001 versus only prednisolone treatment. Statistical tests were performed using one-way ANOVA followed by Dunnett’s Multiple Comparison Test.

In order to assess the in vivo efficacy of tigecycline and minocycline against osteoporosis, we examined the effect of the tetracycline analogs on bone mass in zebrafish larvae. Recent studies have reported that the zebrafish is an ideal model system for the in vivo analysis of GIO [21,22], hence we employed zebrafish larvae for our study. We first tested for the optimal concentration of prednisolone necessary to develop an osteoporosis phenotype. Zebrafish larvae at 10 dpf (days post-fertilization) were treated with various concentrations of prednisolone (0, 5, 10, and 25 μM) for three days, then whole-mount bone staining was performed. Prednisolone had little effect on larvae survival, regardless of concentration. However, we observed that bone mineralization was severely decreased in a dose-dependent manner (data not shown). The most severe bone loss in zebrafish larvae was seen with 25 μM prednisolone treatment; we therefore used this concentration to examine the anti-osteoporosis activity of tigecycline. After simultaneous treatment with prednisolone, with or without the tetracycline analogs, zebrafish bone mineral density was measured. As shown in Figure 5, tigecycline or minocycline treatment gradually reduced prednisolone-induced osteoporosis in a dose-dependent manner.

## 3. Discussion

There is a growing interest in epigenetic mechanisms that regulate gene transcription at various stages of osteoclast differentiation. Although MMP-9 is highly expressed during osteoclastogenesis, and is involved in RANKL-induced osteoclast differentiation, the exact mechanisms how MMP-9 stimulates osteoclastogenesis are unclear. We recently reported that MMP-9 localizes in the nucleus and facilitates active transcription states of osteoclastogenic genes by proteolytically cleaving H3NT. We also showed that both H3K18ac and H3K27me1 are required for MMP-9 to localize and initiate H3NT proteolysis at the genes encoding osteoclast differentiation [6]. Since MMP-9 is a key regulator of osteoclast formation, and is heavily implicated in osteoporosis [23], it is reasonable to develop anti-osteoporosis drugs targeting MMP-9. As tetracycline derivatives are known to be the most common inhibitors of MMP-9, and have also shown differential inhibitory activity [24,25], we first checked their binding affinity for MMP-9, using a molecular docking program. Tigecycline and minocycline were chosen for our study based on their binding energy and therapeutic potential. We clearly showed that exposure of OCP-induced cells to two tetracycline analogs significantly decreased H3NT proteolysis under RANKL-activated differentiation. In line with the ex vivo results, our in vitro cleavage assay also showed that those inhibitors apparently blocked MMP-9-mediated H3NT proteolysis. However, there was a large discrepancy in IC50 values: the ex vivo IC50 = 2.5 μM, and the in vitro IC50 = 50~100 μM. A recent study has observed a similar phenomenon to our study [20]. One possible explanation for the inconsistency may be due to the difference of the enzyme quantity. Although MMP-9 accumulates in the nucleus during osteoclast differentiation, the total amount of nuclear MMP-9 might be much less than that of MMP-9 used for in vitro cleavage assay. Notably, the impairment of MMP-9-dependent H3NT cleavage by MMP-9 inhibitors decreased the number of multinucleated osteoclasts, and suppressed the transcription of osteoclastogenic genes. To our knowledge, this is the first report to describe that tigecycline and minocycline exert their inhibitory effects on osteoclastogenesis by modulating MMP-9 enzymatic activity toward H3NT.

An intriguing finding from our study is that tigecycline and minocycline not only inhibit MMP-9 proteolytic activity, but also significantly repress MMP-9 mRNA expression and protein levels at higher doses (5 μM) (Figure 3, Figure S3). Recently, Ma et al. have shown that cis-acting elements in the MMP-9 promoter include activator protein 1 (AP-1) and nuclear factor kappaB (NF-κB) binding sites, and that the activation of the extracellular signal-regulated kinase (ERK) and NF-κB pathways induces MMP-9 gene expression [26]. In our study, we observed that higher doses of two tetracycline analogs (2.5 and 5 μM) significantly inhibited *c-Fos* gene expression, but not NF-κB expression (Figure 3). These results suggest that the decreased level of *c-Fos* gene expression caused by tigecycline and minocycline may contribute to *Mmp-9* gene expression. Additional studies are needed to understand the exact mechanism underlying the effect of tigecycline and minocycline on MMP-9 expression.

Because tigecycline and minocycline efficiently suppressed osteoclastogenesis, we expected these antibiotics to be effective for osteoporosis treatment. GIO is a serious side effect of glucocorticoid therapy, and can lead to vertebra fractures. Recent studies have shown that treatment with glucocorticoids, such as prednisolone, produce an osteoporosis phenotype in zebrafish larvae [21,22]. Notably, we found that prednisolone treatment induced severe bone loss, whereas tigecycline and minocycline ameliorated bone loss in a zebrafish GIO model. It has been reported that glucocorticoids stimulate the proliferation and differentiation of human osteoclast precursors [27]. Rauch et al. revealed that glucocorticoids suppress bone formation by attenuating osteoblast formation [28]. Based on our findings that tigecycline and minocycline specifically inhibit osteoclast formation, but have less of an effect on osteoblastogenesis, we strongly suggest that tigecycline prevents glucocorticoid-induced bone loss by suppressing osteoclast formation.

Taken together, we demonstrated that tigecycline and minocycline inhibit MMP-9 proteolytic activity toward the H3 tail. We have revealed that the treatment of tigecycline and minocycline suppresses the expression of osteoclast-specific genes by modulating MMP-9-mediated H3 cleavage, thereby influencing osteoclast formation. Finally, prednisolone treatment caused vertebral bone loss in zebrafish, whereas coadministration of tigecycline and minocycline prevented the adverse effects of glucocorticoid use. Our results suggest that tigecycline and minocycline may be of great therapeutic value in treating GIO.

## 4. Materials and Methods

### 4.1. Plasmid Construction and Materials

Histone H3 and MMP-9 (aa 115–730) proteins were expressed in *Escherichia coli* Rosetta 2 (DE3) pLysS cells (Novagen, Madison, WI, United States) and purified from inclusion bodies, as described recently [29,30]. The minocycline and tigecycline were from Sigma-Aldrich (St. Louise, MO, United States). The antibodies used in this study were as follows: H3 C-terminal antibody from Abcam (Cambridge, United Kingdom); MMP-9 antibody from Millipore (Burlington, MA, United States); and NFATc1 and Actin antibodies from Santa Cruz Biotechnology (Santa Cruz, CA, United States).

### 4.2. Docking Simulation of MMP-9 with Tetracyclines

The crystallographic structure MMP-9 (PDB: 4H3X) [31] was retrieved from the Protein Data Bank (https://www.rcsb.org/). All chemicals were downloaded in mol format from ChemSpider Chemical Database. These files were converted into pdb files using PyMOL structure visualization software (https://pymol.org/). All hydrogen atoms were added, and the MMP-9 protein coordinates were then minimized with MOE using the AMBER10 force field, until the root mean square (RMS) gradient of the potential energy was less than 0.1 kJ mol^−1^ Å^−1^. A site finder model was used to identify possible ligand-binding pockets within the newly generated three-dimensional (3D) structures of the ligand–receptor complex. The docking and analysis of the ligand–receptor interaction between ligand and receptor were also performed with the Triangle Matcher method in the MOE.

### 4.3. Osteoclast Differentiation and H3NT Cleavage Analysis

Osteoclast precursor cells were prepared as recently described [6]. Osteoclast precursor cells were cultured in 48-well plates with 30 ng/mL of macrophage colony-stimulating factor (M-CSF) and 100 ng/mL of receptor activator of nuclear factor kappaB ligand (RANKL), in the absence or presence of tigecyclin or minocycline. On day three, the cells were fixed with formaldehyde and stained for tartrate-resistant acid phosphatase (TRAP), using an acid phosphatase leukocyte kit (Sigma-Aldrich, St. Louise, MO, United States). TRAP-positive, multinucleated cells containing three or more nuclei were counted as osteoclasts under a light microscope. To determine the levels of H3NT proteolysis, nuclei were isolated from cells in buffer A (10 mM HEPES, pH 7.4, 10 mM KCl, 1.5 mM MgCl2, 0.34 M sucrose, 10% glycerol, 1 mM DTT, 5 mM β-glycerophosphate, 10 mM NaF, protease inhibitors, and 0.2% Triton X-100), and chromatin was extracted in buffer B (3 mM EDTA, 0.2 mM EGTA, 1 mM DTT, 5 mM β-glycerophosphate, 10 mM NaF, and protease inhibitors). Western blot analysis was performed using an H3 C-terminal antibody, as previously described [6]. Additionally, to evaluate the expression level of NFATc1, MMP-9, and Actin, cells were lysed with lysis buffer (25 mM Tris, pH 7.9, 150 mM NaCl, 0.5% NP-40, 1 mM EDTA, 5% glycerol, and protease inhibitor cocktails), and the lysates were subjected to Western blot analysis.

### 4.4. In Vitro H3NT Cleavage Assays

H3NT proteolysis assays were performed as previously described [6], except that MMP-9 (100 ng) was pre-incubated with the indicated concentration of tigecycline or minocycline for 30 min, and then 1.25 μg of histone H3 was added. After 1 h reaction, H3NT cleavage was determined by Western blotting with an H3 C-terminal antibody.

### 4.5. Osteoblast Differentiation and Alizarin Red S Staining

Primary osteoblasts were isolated from newborn pups, as previous described [32]. Osteoblast precursor cells in 12-well plates were treated with 50 μg/mL ascorbic acid and 10 mM β-glycerophosphate (β-GP), in the absence or presence of tigecycline or minocycline. On day 14 or 19, ARS staining was performed with 2% ARS (pH 4.2), after fixation in 4% paraformaldehyde. For quantifying ARS staining, the stain was solubilized with 10% cetylpyridinium chloroide for 1 h, and the absorbance was measured spectrophotometrically at 570 nm.

### 4.6. RT-qPCR

The total RNA was isolated from osteoclasts using Tri-RNA reagent (Favogen Biotecnology, Ping-Tung, Taiwan), and reverse-transcribed using the Moloney Murine Leukemia Virus (M-MLV) reverse transcriptase (Promega, Madison, WI, United States) and IQ SYBR Green SuperMix (Bio-Rad, Hercules, CA, United States). The sequences of primers used for qPCR were as follows: *Nfatc1* 5′-CTCGAAAGACAGCACTGGAGCAT-3′ (forward) and 5′-CGGCTGCCTTCCGTCTCATAG-3′ (reverse); *Oscar* 5′-CTGCTGGTAACGGATCAGCTCCCCAGA-3′ (forward) and 5′-CCAAGGAGCCAGAACCTTCGAAACT-3′ (reverse); *Ctsk* 5′-ACGGAGGCATTGACTCTGAAGATG-3′ (forward) and 5′-GGAACCACCAACGAGAGGAGAAAT-3′ (reverse); *Trap* 5′-CTGGAGTGCACGATGCCAGCGACA-3′ (forward) and 5′-TCCGTGCTCGGCGATGCACCAGA-3′ (reverse); *p65* 5′-GGAGTTCCAGTACTTGCC -3′ (forward) and 5′-GTCCTTTTGCGCTTCTCT-3′ (reverse); *c-Fos* 5′-CCAGTCAAGAGCATCAGCAA-3′ (forward) and 5′-AAGTAGTCGCAGCCCCGAGTA-3′ (reverse); *Mmp-9* 5′-CGTCGTGATCCCCACTTACT-3′ (forward) and 5′-AACACACAGGGTTTGCCTTC-3′ (reverse); *Lif* 5′-AGAAGGTCCTGAACCCCACT-3′ (forward) and 5′-GCCTGGACCACCACACTTAT-3′ (reverse); *Xpr1* 5′-AGGAGCGTGTCCAACATAGG-3′ (forward) and 5′-CCACGAGATGTTTCCAGGAT-3′ (reverse); *ALP* 5′-GTTGCCAAGCTGGGAAGAACAC-3′ (forward) and 5′-CCCACCCCGCTATTCAAAC-3′ (reverse); *Runx2* 5′-TTCTCCAACCCACGAATGCAC-3′ (forward) and 5′-CAGGTACGTGTGGTAGTGAGT-3′ (reverse); *BSP* 5′-AAGCAGCACCGTTGAGTATGG-3′ (forward) and 5′-CCTTGTAGTAGCTGTATTCGTCCTC-3′ (reverse); and *Osteocalcin* 5′-GCAATAAGGTAGTGAACAGACTCC-3′ (forward) and 5′-GTTTGTAGGCGGTCTTCAAGC-3′ (reverse).

### 4.7. Cell Proliferation Assay

To examine the effect of tigecylcine or minocycline on cell proliferation, osteoclast and osteoblast precursor cells were treated with the indicated concentration of tigecycline or minocycline (1.0, 2.5, and 5.0 M) in 96-well plates, and MTT assays were performed after 24, 48, or 72 h using Cell Proliferation KIT 1 (Roche Diagnostics, Mannheim, Germany).

### 4.8. Fish Maintenance and Drug Treatment

Zebrafish (wild-type strain) were raised under standard maintaining conditions in a circulating water system at 28 °C, with day–night (14 h light/10 h dark) cycles. The fish were fed live brine shrimp three times a day. The male and female zebrafish with high potential to produce fertilized eggs were chosen for spawning. After removing the unfertilized eggs, embryos were kept at 28 °C, with day–night (14 h light/10 h dark) cycles. At 10 dpf, the larvae were treated with 25 μM prednisolone ± three different concentrations (10, 25, and 50 μM) of tigecycline or minocycline. At 13 dpf, the larvae were collected for whole-mount skeletal staining.

### 4.9. Whole-Mount Skeletal Staining

Alizarin red staining on zebrafish larvae was performed as previously described [33]. Briefly, larvae at 13 dpf were fixed overnight at 4 °C in 10% neutral buffered formalin, and rinsed with tap water several times. After bleaching pigmentation with 3% H_2_O_2_ solution, the larvae were stained with 1 mg/mL alizarin red stain/1% KOH, and then sequentially washed with 20% glycerol/1% KOH, 40% glycerol/1% KOH, and 60% glycerol/1% KOH. Images of stained larvae were obtained using a stereo microscope (SMZ18) (Nikon, Tokyo, Japan). For quantification of bone mineral density, the areas of the first four stained vertebrae (V1–V4) were calculated using the ImageJ densitometry program.

### 4.10. Statistical Analysis

All quantitative data are presented as mean ± SD. GraphPad Prism (GraphPad Software Inc. La Jolla, CA, United States) was used for all analyses. Statistical analysis was done using the one-way ANOVA test, followed by Tukey’s Multiple Comparison Test or Dunnett’s Multiple Comparison Test.

## Supplementary Materials

The following are available online at https://www.mdpi.com/1422-0067/20/16/4038/s1.

## Abbreviations

RANK	Receptor activator of nuclear factor kappaB
RANKL	Receptor activator of the NF-κB ligand
MMP	Matrix metalloproteinase
BMM	Bone marrow-derived macrophage
GIO	Glucocorticoid-induced osteoporosis
TRAP	Tartrate-resistant acid phosphatase
ALP	Alkaline phosphatase
MTT	3-(4,5-Dimethylthiazol-2-yl)-2,5-Diphenyltetrazolium Bromide
Runx2	Runt-related transcription factor 2
ALP	Alkaline phosphatase
BSP	Bone sialoprotein
HEPES	4-(2-hydroxyethyl)-1-piperazineethanesulfonic acid
DTT	Dithiothreitol
EDTA	Ethylenediaminetetraacetic acid
EGTA	Ethylene glycol tetraacetic acid
