# Supplementary material for: Tetracycline Analogs Inhibit Osteoclast Differentiation by Suppressing MMP-9-Mediated Histone H3 Cleavage
Source: Int J Mol Sci. 2019 Aug 19;20(16):4038. doi: 10.3390/ijms20164038 (PMC6719029; doi:10.3390/ijms20164038)
Supplement: Supplementary file 1 [file ijms-20-04038-s001.pdf]

Tetracycline analogs inhibit osteoclast differentiation by suppressing MMP-9-mediated histone H3 cleavage

Yeojin Kim, Jinman Kim, Hyerim Lee, Woo-Ri Shin, Sheunghun Lee, Jisu Lee, Jae-Il Park, Byung Hak Jhun, Yang-Hoon Kim, Sun-Ju Yi, and Kyunghwan Kim

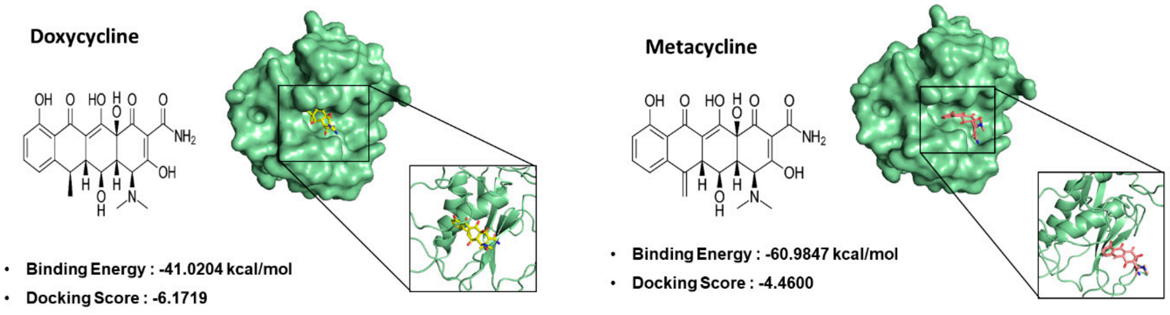

Figure S1. Molecular docking analysis of MMP-9 and tetracycline derivatives.

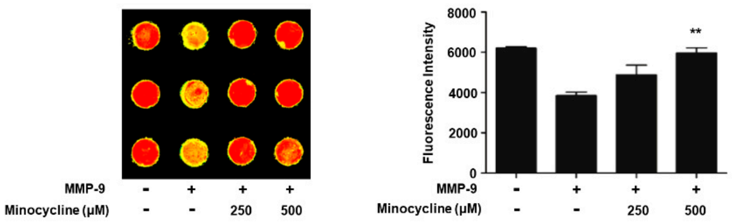

Figure S2. Peptide chip assay. Peptide arrays were fabricated by immobilizing TAMRA-Histone H3 peptide onto well-type amine array. Histone cleavage assays were performed as in Material and methods. Results are obtained from three independent experiments. Bars, the mean result ± S.D. $^{**}p < 0.001$ relative to H3 peptide treated with only MMP-9.

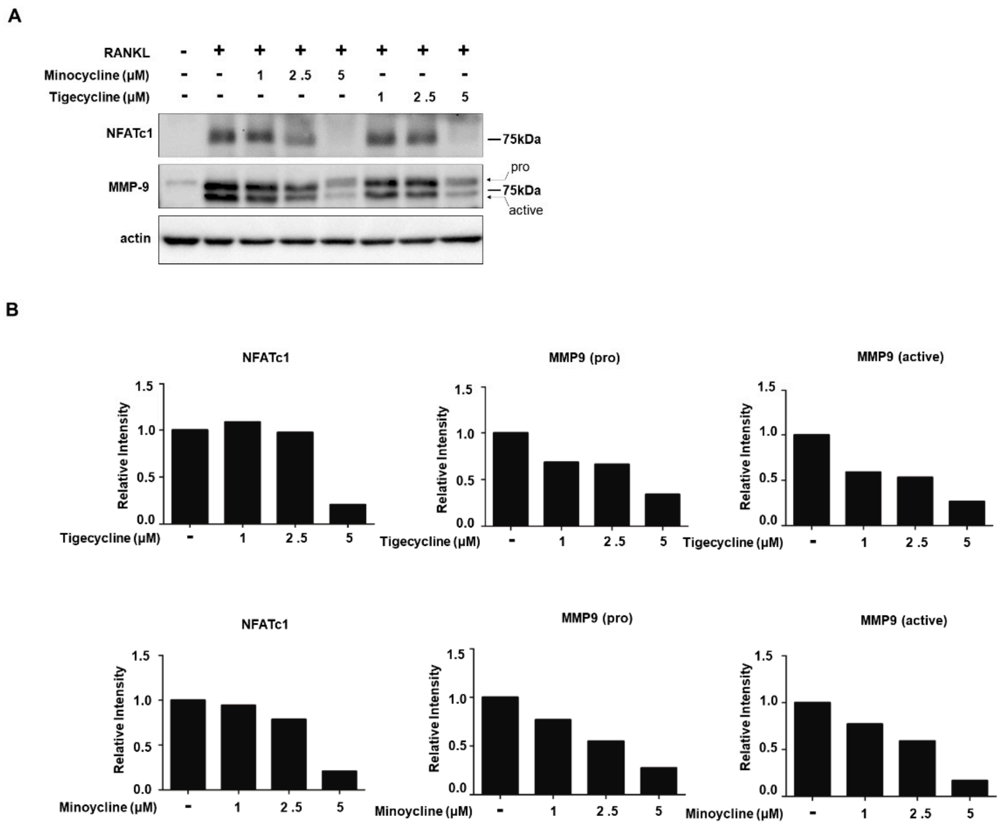

Figure S3. Western blot analyses of NFATc1 and MMP-9 after treating with OCP cells with tigecycline and minocycline. (A) BMM cells were pretreated with tigecycline or minocycline and further with RANKL for 3 days. Whole cell lysates were prepared and analyzed by Western blotting with the indicated antibodies. β-actin was used as a loading control. (B) The band intensities of the Western blots were quantified by densitometry.

Supplementary Materials and Methods

Peptide chip analysis

Well-type amine arrays were prepared by attaching Teflon tape on to silane-coated glass slides (Muto Pure Chemicals, Tokyo, Japan). To make well-type amine arrays containing TAMRA-labeled Histone H3 (aa 14-28), 4.9 mM sulfo-GMBS (ThermoFisher Scientific, Waltham, MA, USA) were dropped into 1.5mm tape hole. After 2h incubation, the arrays were washed with 50 mM sodium bicarbonate buffer (pH 7.0). Then, 1μM of H3 peptide were applied to array wells and further incubated at 37 °C for 1h. To investigate the effect of minocycline on MMP-9-mediated H3 proteolysis, the mixture of MMP-9 with or without minocycline was applied to TAMRA-Histone H3 peptide array and incubate at 37 °C for 1h. Following washing with 0.1% Tween 20 in phosphate buffered saline and with milli-Q-purified water, the arrays were dried and scanned on a GenePix 4000B (Molecular Devices, San Jose, CA, USA) scanner using 532 nm lasers.
